# Elements of Materia Medica and Therapeutics; Including the Recent Discoveries and Analyses of Medicines

**Published:** 1834-04-01

**Authors:** 


*Elements of Materia Medica and Therapeutics; including the recent Discoveries and Analyses of Medicines.* By Anthony Todd Thomson, m.d. Vol. II.—London, 1833. 8vo. pp. 694.

Dr. A. T. Thomson is well known to our readers as the author of a Dispensatory which is in everybody's hands, and which unquestionably entitles him to rank as our best writer on the Materia Medica. In the present work (of which only the second volume is before us,) the articles are not arranged alphabetically, as in the Dispensatory, but in classes, whose limits are marked out by their therapeutic power, as the Emetics, the Cathartics, &c. The general mode of action of each class is illustrated by pretty copious remarks; and, moreover, our author has not confined himself so strictly, as in his former work, to the substances enumerated in the three British Pharmacopœias.

Everything on the Materia Medica which proceeds from Dr. Thomson must have some merit, and our readers will thank us therefore for a notice of the "Elements," confined chiefly to such points as we believe are not touched upon in the "Dispensatory." The following extracts from the chapter on Astringents are not without interest:

"As an astringent, the Sloe was employed in the time of Dioscorides, and is still used as a domestic remedy, although it has been rejected from the Pharmacopœias. It has one advantage over many other substances in this class of remedies: it exerts no stimulant influence, and therefore may be administered even when inflammatory symptoms exist. It has been recommended in diarrhœas, especially in those of a chronic character, such as are often brought on in India: in hæmorrhages, and as a topical astringent in enlargements of the tonsils and relaxation of the uvula. It was formerly administered as a conserve; but the inspissated juice of the unripe fruit, or a tincture in proof spirit, is preferable. The inspissated hardened juice may be given in powder, in doses of from eight grains to a scruple, three or four times a day." (P. 34.)

"*Ruspini's Styptic*, one of those medicines which are known by the name of Patent, the preparation of which is kept secret, and which are often little more than frauds of designing knaves on the credulity of the public, owes its powers to gallic acid. Whilst we may declaim against the principle which has withheld the formula from the public, we cannot deny the value of the preparation as a powerful astringent. I have not witnessed its influence as an internal or general astringent; but I have frequently witnessed its power in checking the most obstinate bleedings from leech-bites in children, after all other things had failed. This styptic consists of gallic acid, a small proportion of sulphate of zinc and of opium, dissolved in a mixture of alcohol and rose-water. In proof of this, the same re-agents which affect Ruspini's styptic, affect, in the same manner, a simple solution of gallic acid in alcohol. It yields a brownish-green precipitate with lime-water, which is re-dissolved by an excess of the lime-water, and acquires a reddish hue: and it strikes a beautiful deep blue with the mixed sulphates of iron. As the quantity of sulphate of zinc and of opium is too small to influence the medicine, a simple solution of gallic acid in diluted alcohol will answer all the purposes of this celebrated and expensive styptic." (P. 47.)

We are surprised that our author should have been obliged to have recourse to this styptic *frequently* in the cases of leech-bites, as the bleeding, if it does not yield to cold water, can almost always be stopped by a minute portion of sponge fastened upon the wound with sticking-plaster.

In administering the acetate of lead, Dr. Thomson insists on the advantage to be derived from giving vinegar at the same time; for the carbonate is the only salt of lead which is directly poisonous, and the vinegar prevents the decomposition of the acetate in the intestines. He also refers to Mr. Laidlaw's experiments, to which we directed the attention of our readers on a former occasion. (Vol. i. page 120.) We cannot quit the subject of lead without reminding them of the masterly researches of Dr. Christison, detailed in his work on Poisons, page 385 et seq.

The following observations are characterized by the good sense which pervades our author's practice.

"In hæmorrhoids, the propriety of employing astringents depends altogether on the remote causes of the disease. The most common of these is a confined state of bowels; thence purgatives, or rather laxatives, are indicated; when there is heat, hardness, and much pain, leeches should be applied; but after these symptoms are removed, or where they are not present, when the piles are large and the bleeding excessive, then astringents should be employed. A pint of cold water thrown into the rectum every morning, by means of a gum elastic bag, an ointment composed of powdered gall nuts, or of kino or catechu and lard, or solutions of the metallic salts, such as sulphate of zinc in solution of alum, may be administered. When hæmorrhage proceeds from a ruptured vessel high up in the rectum or in the colon, the stomach-pump should be used, either to throw in cold water, or infusions of the astringent vegetable bodies, or solutions of the saline astringents. Whatever be the nature of the astringent solution or infusion, the quantity should not be such as to irritate by distention, or to cause too rapid an evacuation of the injected fluid. Accompanying this state of the hæmorrhoidal vessels, we not unfrequently find prolapsus ani, or falling down of the fundament. This also occurs occasionally in children and in old people, from mere debility, on the slightest effort to relieve the intestines of their contents. The return of the gut in this state is easily affected; but it is only by bracing and invigorating the loose and relaxed membrane that we can expect it to remain in its proper place. This is best accomplished by astringent injections; and nothing is so well adapted for these as the infusion of the pomegranate-bark, or that of balaustines.

" Hæmaturia, or bleeding from the bladder, is generally depending upon some organic affection of the urinary organs; but, in attending to the primary disease, much immediate advantage is derived from the use of astringents. It was in a case of this kind that Mr. Brodie administered Ruspini's styptic with so much seeming advantage; and I have seen great benefit, in similar cases, from the use of uva ursi, which appears to pass unaltered through the kidneys. Since the discovery which I have made of the composition of Ruspini's styptic, I am disposed to propose a combination of gallic acid with an infusion of the leaves of uva ursi, obtained by rubbing them in cold water." (P. 86.)

The difference between precept and practice has often been exemplified: take another instance. " An amusing fact, connected with the opposition to the general use of tobacco, is related of Fagou, physician to Louis XIV. In the midst of an oration on its pernicious effects, the orator made a pause, and, taking his snuff-box from his pocket, refreshed himself with a pinch, to enable him to renew his argument." (P. 120.)

Our author's remarks on the inhalation of chlorine will be read by many with advantage.

" *Chlorine.* This gas is of very late introduction as an expectorant and excitant of the diseased mucous membrane of the lungs.

" Chlorine gas is rapidly absorbed by water; and in this form it may be preserved for extricating the gas for expectorant purposes; but it must be kept in a blackened bottle, as the water is slowly decomposed, and chloric and muriatic acids are formed. Its goodness is known by mixing the solution with a little tincture of litmus: if the solution be good, the colour of the litmus will be totally destroyed; if it contain chloric or muriatic acid, the litmus will be reddened.

"Chlorine, if attempted to be breathed in its undiluted state, does not enter the lungs, but produces a powerful spasm of the glottis; and, if not immediately relieved, the person dies of suffocation. When diluted with a moderate portion of air, it excites violent coughing, irritation in the bronchial cells, great dyspnœa, and a painful, anxious sensation in the chest, which continues for several days. Yet, when largely diluted, this gas is the best topical expectorant and the most salutary excitant to the mucous membrane of the lungs that has yet been inhaled. I have witnessed its beneficial effects in spasmodic cough and in asthma; and I have seen much benefit result from its cautious employment even in phthisis pulmonalis.

"This gas, in its highly diluted state, was first proposed to be employed as a pneumatic remedy and an expectorant by Dr. Favart, of Marseilles, in 1804. His explanation of its action is, 'that by irritating in a peculiar manner the mucous membrane, it draws towards that membrane the matter gorging the pulmonary parenchyma; and, thus rendering it susceptible of evacuation, it relieves the lungs in severe catarrh.' It is unnecessary to comment on the improbability of this hypothesis. Soon after this period, I had an accidental opportunity of witnessing its beneficial influence in a severe case of epidemic catarrh, in which it was extricated as a fumigation to check infection; but it was not employed either on the continent, or in this country, by more than one or two physicians, until lately, when a report of Dr. Cottereau, to the Faculty of Medicine of Paris, again brought it before the profession.

"Several trading chemists, and in particular a M. Gannal, had remarked that phthisical persons, who engage themselves to work in the manufactories of bleaching liquor, and other processes, in which chlorine is extricated, are gradually but evidently improved in health; and, to confirm his observations, he constructed an instrument for inhaling it, and actually administered it as a remedy in phthisis. The success of the experiment surprised M. Gannal; but, not being a medical man, he mentioned his views to Dr. Cottereau, who pursued the same plans as M. Gannal, and with a degree of success sufficient to merit the attention of the profession. As far as my own experience enables me to offer an opinion, chlorine will form a most valuable auxiliary in the treatment of chronic catarrh, humoral asthma, and even phthisis. In the two former diseases, I have relieved several individuals by its means; and, in all the cases of phthisis in which I have employed it, the benefit has been sufficient to encourage the most sanguine expectations of success; but none of the cases have been cured.

"For the purposes of inhaling, chlorine should be extricated from the saturated aqueous solution of the gas, by putting f℥i or f℥ii of it into a tubulated bottle, containing about f℥ii of hot water, and placed either in a bason of hot water or over a small lamp, in order to extricate the chlorine from its aqueous solvent. The patient should inhale this quantity at one time, and the dose should be repeated once, at least, every six hours, so as to maintain the effect produced on the mucous membrane.

"When thus cautiously inhaled, the evident effect of chlorine, is, at first, a slight sense of constriction in the trachea and some increase of cough: in a few instances, a degree of vertigo has been experienced and a tightness across the chest; but these feelings rapidly subside, expectoration is produced almost without any effort, and the patient feels generally more comfortable than before inhaling the gas. In those cases of asthma, in which I have seen the chlorine used, the relief is peculiarly striking; and even in phthisis, the symptoms of hectic have much abated during its employment; so that in cases in which a fatal termination of the disease has occurred, the chlorine may be said to have 'scattered flowers on the borders of the grave.' Its operations can only be explained on its stimulant power, producing a new action in the morbid organ; which, if it could be maintained sufficiently long, might assuredly overcome the diseased action; and by the assistance of other means calculated to support the tone of the habit, without exciting fever, the disease might be cured. In cases where large vomicæ exist, it is in vain to expect a cure from any means; but when we consider the powerful influence of chlorine in checking putrefaction and in promoting the cure of external ulceration, it is not, in my opinion, a vain speculation to expect advantage in ulcerated lungs from this mode of employing chlorine.

"Like every other powerful gaseous excitant, chlorine, when inhaled without being sufficiently diluted, produces a severe sensc of strangulation, a violent irritating cough; and individuals have fallen down in a state of complete syncope who have suddenly taken a large draught of it. These effects, however, are only temporary; and few instances have occurred in which inflammation of the lungs and air-tubes have supervened. Indeed to no other gas does the system so soon accommodate itself; the workmen in chemical manufactories breathe it daily with impunity.* The best method of overcoming its deleterious effects is to inhale the vapour of ammonia diluted with water or to inhale ether; or, if neither of these substances be at hand, to hold the head over a bason of boiling water, so as to breathe the warm vapour." (P. 149.)

In the note at p. 156, Dr. Thomson says, "It is pleasing to trace the origin of popular customs: that of smoking is unknown; but all the Scythian nations employed certain herbs, which they threw into the fire, and the ascending smoke of which the company seated round the fire collected, causing them to dance and sing." In a note upon this note, we are referred to Herodotus, lib. i. § 36, as the authority; but, on turning to the book and chapter, as directed, we

* "Mr. Tenant, one of the greatest manufacturers of bleaching liquor, has informed me that men affected with chronic cough, who apply to him for work, invariably lose their coughs when they are cautiously brought into the gas-house."

found, without much surprise, that there was not a word about the matter there. We say without much surprise, for our author, though excellent in other points, is by no means happy in quotations and references. Thus, at page 302 we find "Ideoque, (says Celsus,) omnibus catharticus aloë miscenda est." Now, without stopping to scold the doctor for cathartic*us*, we must mention that Milligan's Index (a most capital one,) gives no hint of such a passage. At page 242, *note*, we have a bit of Boerhaave so cruelly defaced, that it is difficult to transcribe it, without correcting some of the errors: "Oportet quidam, hic monere quod leniara emetica nil agunt in Ascite, sed fortiera ex brevibus intervallis repetita palmam reliquis præripiant. Boerhaave, in *Pract. Med. Ars.*, art. *Hydrops.*" These, and a myriad of other slips of the pen and of the press, would be of small importance, were the work destined only for the man of education; but a book of the kind is consulted by the raw beginner, who is naturally misled by errors which bear the professorial stamp.

There has always been much diversity of opinion as to the plant which yields gum ammoniacum. "Willdenow, who had raised an Heracleum from seeds picked from ammoniacum, was induced to regard it as a species of that genus, and named it Heracleum *gummiferum;* Sprengel asserted that it was the Ferula *ferugala* of Defontaines; Olivier, that it was the Ferula *Persica;* whilst others contend [contended] that the plant was the Bubon *gummiferum* of Linnæus: in one point only all agreed, viz. that ammoniacum is the product of an umbelliferous plant." (P. 163.) It seems, however, that all these learned persons were in the wrong, and that this long-established expectorant is derived from a newly-discovered plant, which has been named by Mr. Don, Dorema *Ammoniacum.* Mr. Don says, too, "that the plant which affords Galbanum is not the Bubon *Galbanum* of Linnæus, but one which appears to constitute a new genus allied to Siler, but differing from it in the absence of dorsal resiniferous canals, and the commissure being furnished with only two. He proposes to call the plant Galbanum Officinale." (P. 165.)

Our author asserts, and he is quite orthodox in his assertion, that "emetics ought not to be administered to those afflicted with *hernia*," (p. 197;) but we have strong doubts of the universal, or even general force of the objection: one sixth or one eighth of his Majesty's subjects ought not to be deprived of the benefits flowing from this strangely neglected class of remedies, when a little extra pressure on the abdominal parietes will assuredly guarantee their safety. Indeed, the hand of the patient, which in such cases is often instinctively applied, is an excellent temporary truss. Do not the ruptured go to sea?

Our author, at p. 205, quotes the testimony of Dr. Young in favour of the use of emetics in phthisis, and such is the carelessness with which modern works are put together, that the same quotation occurs again at p. 238. Dr. Thomson mentions the influence of emetics in relieving gastric amaurosis, but has omitted to notice their unrivalled power of relieving many forms of dyspepsia. It often happens, for instance, that among the Protean symptoms attending that shapeless monster, dyspepsia, we find a rising of bile in the mouth: in such a case, an emetic stands alone in efficacy; and, if there are many diseases which are justly termed the *opprobria medicinæ*, a case of *dyspepsia biliosa*, instantaneously relieved by an emetic, may be considered as a splendid triumph of the healing art.

Nor are we quite sure that in fevers it is necessary so rigorously to limit the use of emetics to the commencement of the attack. Take the following case, for example, which we find among our notes. "June 29th, 1833. Mrs. J., ætat. twenty-four, was attacked with influenza three weeks ago; since which time she has suffered from nausea, want of appetite, and general pains: tongue white, pulse rapid.—℞. P. Scammon. c. ℈j.; Antimon. Tart. gr. j. M. ut ft. pulv. statim sumend.—Half-past eight, P.M. The powder has produced two stools, and frequent vomiting. The quantity of bile brought up is enormous. The patient feels quite comfortable.—June 30th. Her bowels have been open; she has perspired freely, and has slept well; the appetite has returned, there is no nausea, and she feels 'quite charming.' Cured." Had this case been treated by a calomelarian, it certainly would not have been cured in twenty-four hours; and, had some ingenious person found out that Mrs. J. was labouring under "chronic gastritis," and applied his leeches, and administered his *eau gommée*, she might have been under treatment at this very hour.

It is so difficult to know under what head many medicines should be classed, that we do not find fault with Dr. Thomson for placing copaiba among the cathartics, though it is rarely administered as a purgative; and our readers will certainly be gratified by some of our author's observations on this remedy.

"The action of re-agents enables us to ascertain the purity of copaiba. The simplest mode is to boil any given quantity of the balsam in water to dryness; if the copaiba be pure, a hard, brittle resin will remain; thence the consistency of this residue determines the purity of the specimen. Another simple method is to mix two parts of copaiba with one part of an alkaline solution, consisting of three fourths of carbonate of potassa and one of pure potassa: if the copaiba be pure, after some hours the mixture divides into two parts: but if the balsam is adulterated with one eighth of castor oil, the whole will remain as a gelatinous mass. If four parts of copaiba and one of carbonate of magnesia be rubbed together and left at rest, it will assume an appearance not unlike solution of gum acacia, if the copaiba be pure; but it will be opaque, if the copaiba be adulterated with oil. There are other tests; but these are sufficient to determine the purity of this substance.

"Copaiba is either a simple stimulant or a purgative, in proportion to the extent of the dose. In doses of ʒj, rubbed into an emulsion with mucilage of gum, it operates kindly on the intestinal canal, and affords great relief of hæmorrhoidal affections; both evacuating the contents of the rectum and allaying the irritability of the inflamed surface, by lessening the determination of blood to the part. This effect may probably be in part owing to the determination which it induces to the kidneys, therefore operating as a counter-irritant. There are various methods of administering the remedy: among others, by spreading a pound of copaiba on a dish and sprinkling over it an ounce of calcined magnesia, then mixing it intimately, and exposing the mixture to the air for fifteen or twenty days, it acquires a consistence fit for making pills, which possess the same efficacy as the pure copaiba. The essential oil procured by distillation has the same properties as the copaiba; but the resin is inert.

"When the copaiba or the oil is moderately overdosed, it sets up fever in the system, accompanied with headach, thirst, great heat in the bowels, and a sensation of burning in the urethra while passing the urine; and the kidneys are so much stimulated that bloody urine is secreted. But, like some other oleo-resins, these symptoms do not occur when the dose is so large as to operate at once upon the bowels. A French officer at Valladolid, in 1808, took two ounces of copaiba for a dose; it operated as a drastic cathartic, and cured a gonorrhœa, under which he was labouring, without causing much inconvenience.*" (P. 284.)

A little farther on we find a notice of *Jalapine*.

"Mr. Hume, an intelligent chemist in Long Acre, obtained a substance from jalap by an operose process; and, regarding it as the active principle of jalap, he named it *Jalapine*. Only five grains are obtained from an ounce of the root: it has neither taste nor odour; is scarcely soluble in either cold or hot water; and completely insoluble in ether. No therapeutical trials have been made with this substance; but, from the result of M. Pelletier's experiments with the sulphate of jalapine, sent to him by Mr.

* "Revue Médicale, tome ix., p. 10."

Hume, I am not disposed to regard it as the active principle of jalap." (P. 290, *note.*)

We recollect that several trials were made with jalapine at St. George's Hospital, by that excellent physician, the late Dr. George Pearson; but it certainly was by no means active.

We positively must mention another instance of our author's carelessness; it is really too bad. At page 337, after the words *Gratiola Officinalis*, occur the symbols L.E.D., importing that the plant is in the three British Pharmacopœias; yet in the next page we are told "Gratiola is not contained in the list of the materia medica of the London college."

Our author asserts that elaterium seldom acts well in doses of less than a grain: we have seen a quarter of that quantity, however, purge most violently.

His method of treating tetanus we will give in his own words:

"I cannot avoid taking this opportunity of making public my experience of the powerful influence of cupping along the spine, in relieving the spasmodic rigidity of the muscles in tetanus, in conjunction with the exhibition of cathartic glysters, as long as the jaw remains fixed; and purgatives, combined with opium, on the first moment that any relaxation of the spasm permits their introduction into the stomach. I have had an opportunity of witnessing the successful effects of this plan of treatment in two cases; one of which was a case of traumatic tetanus. Blood was drawn from the cervical portion only of the spine; but dry cupping was employed throughout the whole length of the spine three times a day. The patient informed me that each time the cups were applied, the acute pain under the apex of the sternum was always relieved, and did not return for sometime afterwards." (P. 372, *note.*)

While discussing the therapeutic operation of cathartics, Dr. Thomson also mentions another remarkable instance of their value:

"An interesting case is detailed by Dr. Bostock, in the seventh volume of the Medical Gazette, of a boy having been cured of stammering by purgatives. Dr. Bostock thinks that stammering often depends on a state of certain muscles, resembling that condition of all the muscles in chorea, and which may be termed local chorea. The alvine discharges were offensive and dark-coloured." (P. 374.)

One only of Dr. Lugol's formulæ for the internal administration of iodine is given in the work before us; but, as Dr. Lugol's methods seem to have both theory and practice in their favour, we make no apology for quoting three.

*Ioduretted Mineral Water.*

| | NO. I. | NO. II. | NO. III. |
|---|---|---|---|
| ℞. Iodines . . . . | gr. ¾ | gr. j. | gr. j¼. |
| Potass. Hydriod. | gr. jss. | gr. ij. | gr. ijss. |
| Aq. distill. . . . | ℥viij. | ℥viij. | ℥viij. |

Lugol's formulæ are evidently intended to be swallowed as they are, and not further diluted, as Dr. Thomson directs, (p. 394;) their mildness, and the possibility of continuing their use for many weeks, depend of course on their being already very weak.

Our author ventures to affirm, that the Mist. Ferri comp. is the most injudicious of the preparations of iron.

"It is intended to produce in this preparation a carbonate of iron, suspended in the mixture by the gummy matter of the myrrh: but if the bottle containing it be not quite full and be not kept completely closed, oxygen is rapidly attracted from the air, and the carbonate is as rapidly transmuted into the insoluble and consequently inert peroxide of iron. This is readily demonstrated by the change of colour which takes place in the mixture when exposed to the air. When made at the time it is to be used, however, this mixture is an excellent tonic emmenagogue in doses of f℥iss, given twice or three times a day. The quantity of the protosulphate proper to mix with f℥iss of the mixture of myrrh and carbonate of potassa is four grains. Its influence is perceived by the rapid change which it induces on the alvine and renal evacuations; the black colour of the former, and the blue streak when the latter is tested with ferrocyanate of potassa, demonstrating that the chalybeate has entered the circulation." (P. 464.)

Now, we rather agree with the author of the "London Dispensatory," who says, "This mixture, which is nearly the same as the celebrated antihectic mixture of Dr. Griffith, is an useful tonic in all cases in which preparations of iron are indicated," &c. (*4th edition*, p. 876.)

In speaking of digitalis as an emmenagogue, Dr. Thomson says,

"The influence of foxglove on the generative organs is undoubted. In men, it causes erections and pollutions; in women, it produces symptoms very closely resembling those which indicate the approach of menstruation; and one of the effects of an overdose is inflammation of the genital organs in both sexes. Had foxglove not been employed as an emmenagogue, these facts would be sufficient to authorize its administration for awakening the energy of the uterus. I have long been in the habit of ordering it in doses of from gr. i. to gr. iii. combined with calomel, and followed by an aloetic cathartic on the following morning, with almost unvarying success, in suppression of the catamenia. It is scarcely necessary to say that its use need not be continued many days after the period of the monthly change, and that it is productive of the greatest benefit when it is given, for two or three successive days, anterior to the time when the change should occur. The tincture, for reasons formerly stated, is the best form of administering the medicine." (P. 465.)

It is remarkable that these facts have escaped the notice of Dr. Christison, who merely observes, that "From an extensive series of experiments on animals by Orfila with the powder, extract, and tincture of the leaves, foxglove appears to cause, in moderate doses, vomiting, giddiness, languor, and death in twenty-four hours, without any other symptom of note; but, in larger doses, it likewise produces tremors, convulsions, stupor, and coma. It acts energetically both when applied to a wound, and when injected into a vein." (*A Treatise on Poisons*, p. 633.) It is to the experiments of the Leipsic Society, we believe, that we owe the first account of its stimulant properties, for it was formerly called a *direct* sedative; as, for example, by our author. (*Dispensatory*, 4th edit., p. 321.) Dr. Jörg, the reporter of the Experimental Society, observes, "Es wirkt das Mittel primär erregend auf das Gehirn, auf den Darmcanal, auf die uropoëtischen, und auf die Geschlechtswerkzeuge und secundär herabstimmend auf das Gefässsystem." *Materialien zu einer künftigen Heilmittellehre, &c.* (Leipzig, 1825. S. 468.)—*i. e.* "This remedy acts primarily as a stimulus on the brain, the intestinal canal, the urinary, and the genital organs; and secondarily, as a sedative on the vascular system." And, in the next page, its particular effects as an aphrodisiac and emmenagogue are specially mentioned. It may be worth mentioning, that one of the experimenters took the foxglove without any effect, though he carried the dose as high as ten grains: the medicine was good, and he was seen to swallow it. (*Materialien, &c.* S. 455.)*

Our author writes with the ease and fluency of a man who is discussing a subject with which he is perfectly familiar, and the work consequently reads very pleasantly. Those who confine themselves to a small number of select books may be content with the "Dispensatory;" those who aspire to a library should have the "Elements" likewise.

* Dr. Jörg, in another work, ("Ueber die Kinderkrankheiten,") observes that, by his experiments, he discovered in a few weeks what that excellent physician, Gölis, was sixteen years in ascertaining, namely, the inutility of digitalis in hydrocephalus: indeed, he says, as it is a stimulus to the brain, it must be injurious.

